# Negative life events and college students’ adjustment: the mediating role of self-esteem and the moderating role of grade

**DOI:** 10.3389/fpsyg.2024.1265870

**Published:** 2024-05-02

**Authors:** Xiaoshan Quan, Ji Sun

**Affiliations:** ^1^School of Education, Anshun University, Anshun, China; ^2^Rural Revitalization Research Center of Guizhou Universities, Anshun, China

**Keywords:** negative life events, adjustment, self-esteem, grade, college students

## Abstract

The present research explored the relationship among negative life events, self-esteem, grade, and adjustment of college students. In total, 1,717 college students were investigated by the adolescent life events scale, Rosenberg self-esteem inventory, and Chinese college students’ adjustment scale. The results showed that negative life events were negatively correlated with self-esteem and college students’ adjustment. Moreover, self-esteem was positively correlated with college students’ adjustment. Negative life events significantly negatively predicted college students’ adjustment, and self-esteem mediated the relationship between negative life events and college students’ adjustment. Grade moderated the effects of negative life events on college students’ adjustment. These findings have broad implications for college students’ mental health.

## Introduction

The university period is a critical period in which the individual gradually matures. College students’ adjustment not only affects their learning achievement, physical and mental health, and the development of personality and social function in college (Fan and He, 2018; Sun, 2018) but also has an impact on the future psychological adjustment and development (Wang and Li, 2020; Wang, 2022). College students’ adjustment refers to the individuals’ ability to carry out a comprehensive and objective understanding regardless of the changes in their situation, to adjust their behavior on time according to the changes in the environment, and to keep themselves in a good state (Fang et al., 2005). A study found that only 10% of freshmen did not have problems with adjustment since entering the university, while nearly 50% have adjustment problems in school (Liang, 2013). The detection rate of school maladjustment in freshmen was more prominent than 57.2% (Guo et al., 2014; Li et al., 2016). Some studies have found a relationship between the active withdrawal of college students and maladjustment in school curriculum, teacher teaching, and teacher-student relationships (Guo et al., 2005; Wang, 2009). Individuals cannot deal effectively with college students’ adjustment problems and are prone to negative emotions and behaviors (depression, anxiety, and insomnia) (Cao and Luo, 2020; Zhang et al., 2018). Therefore, it is necessary to explore college students’ adjustment.

This study focuses on the relationship between negative life events and adjustment of college students and elucidates the underlying mechanism of this relationship. As a psychosocial stressor, negative life events are events or situations that challenge, threaten, or exceed individuals’ physical and mental capacity in the living environment, such as family, study, and work (Liu et al., 1997). As a critical factor (Hetolang and Amone-P’Olak, 2018), negative life events are an essential cause of students’ maladjustment (Ma et al., 2020). According to the life history theory, individual’s unique life experience will affect individual behavior patterns and form different life history strategies (Peng et al., 2016; Chang and Lu, 2018). When an individual experiences an extremely harsh or unstable life environment in childhood (negative life events), he or she develops faster life history strategies in early adulthood (Ellis et al., 2009) and is more likely to exhibit behavioral outcomes such as aggressive, antisocial, externalizing problem behaviors and poor academic performance (Chang and Lu, 2018; Fan and He, 2018; Chang et al., 2019; Lu and Chang, 2019). Studies have shown that children’s aggressive behavior has a significant negative predictive effect on peer acceptance and academic achievement. In the process of children’s adjustment to school, aggressive behavior will play a lot of negative effects, resulting in many difficulties in the process (Guo et al., 2005). The frequency of negative life events was significantly negatively related to school adjustment (Liu et al., 2018; Zhang et al., 2018; Wang, 2022), and the higher the score of negative life events, the lower the score of adjustment (Font and Jamie, 2018; Zhang et al., 2018). Studies have also linked negative life events to social functioning (such as morality), showing that people who experience more negative events are more self-centered in moral judgment and reasoning (Zhu et al., 2018). Repeated, long-term or high-impact negative events can weaken one’s beliefs and alter cognition, which may eventually interfere with coping styles and lead to immersive thinking patterns (Wang and Li, 2020). Therefore, we hypothesized that negative life events as external stressors impact college students’ adjustment.

Based on the previous literature, self-esteem is closely related to negative life events and college students’ adjustment. Which may be a mediating variable worth considering. Self-esteem refers to an individual’s emotional experience and evaluation of self-worth formed in the process of socialization (Tian and Li, 2005). Self-esteem includes positive and negative evaluations of the individual (Baumeister et al., 1996). As a positive psychological resource (Zhang et al., 2015; An et al., 2022), self-esteem is an essential protective factor for the healthy growth of adolescents (Ambriz et al., 2012; Li et al., 2019). Negative life events particularly impact adolescent self-esteem (Luciano and Orth, 2016; Sang et al., 2019). There is a significant negative correlation between self-esteem and negative life events (Jiang et al., 2019). Individuals with low self-esteem are susceptible to negative life events (Liu and Liu, 2021). Negative life events negate individuals’ evaluation of their value and ability, thus forming low self-esteem. Some studies found that self-esteem is an important factor affecting individual adaptive behavior (Pan, 2015). Self-esteem is closely related to college students’ adjustment, and individuals with high self-esteem analyze and view themselves positively, cope and solve problems well, and develop well (Baumeister et al., 2003; Song et al., 2020; Christoph and Lea, 2021). There was a significant positive correlation between self-esteem and school adjustment (Li et al., 2021). Moreover, individuals with low self-esteem often underestimate their abilities, have no self-confidence, and often pay attention to and care about their evaluation by the outside world. Once frustrated, they easily feel inferior and harmful, cannot communicate effectively with the outside world, and may be delayed in school adjustment (An et al., 2016; Gao et al., 2020). Some studies further explored the relationship between negative life events, self-esteem and school adjustment, and the results showed that negative life events would have an impact on school adjustment through self-esteem (An et al., 2022). Therefore, we hypothesize that self-esteem plays a mediating role in the relationship between negative life events and adjustment of college students.

As a student progresses in grade (age), their thinking matures. They have their ideas and corresponding ways of dealing with many things. In China, studies have shown that there were significant grade differences in college students’ school adjustment. Specifically, the adjustment scores of freshmen are significantly lower than those of sophomores, juniors, and seniors (Zhang, 2018). Significant differences also exist in the number of negative life events in college (Zhang, 2022). Seniors experience the most negative life events because seniors are facing graduation. At the same time, seniors are about to enter the workplace. It also takes time to adapt from theoretical learning to practical skill operation. In contrast, senior students have fully adapted to school life, accumulated much experience dealing with daily affairs, and can face negative life events more calmly (Zhang, 2022). There is still a process for freshmen to adapt to the new environment. In this process, freshmen tend to be self-centered, easily affected by emotions, and challenging to deal with negative life events. Sophomore students have fully adapted to college life. In the face of stressful events, they have some experience coping with them. While enjoying the beautiful college life, sophomore students also have more free time and are relatively relaxed. The junior year is experiencing the tremendous pressure of obtaining certificates and taking the postgraduate entrance examination and is also sensitive to negative life events (Zhang, 2020). Therefore, we hypothesized that grade has a significant moderating effect on the relationship between negative life events and adjustment of college students.

The present study explored the potential mechanisms underlying the relationship between negative life events and college students’ adjustment. Moreover, we examined the mediating and moderating effects of self-esteem and grade on the relationship between negative life events and college students’ adjustment. It was hypothesized that (1) negative life events are closely related to college students’ adjustment; (2) self-esteem plays a mediating role in the effect of negative life events on college students’ adjustment; (3) grade plays a moderating role in the effect of negative life events on college students’ adjustment.

## Methods

### Participants

Two thousand students from six college schools were recruited to participate in this study. The data of 137 students were excluded owing to the missing data on demographic information and experimental measures. Approximately 49.04% of the students were women, and 50.96% were men. Also, 28.83, 26.44, 23.89, and 20.85% of the students were freshmen, sophomores, juniors, and seniors. The average age of students was 21.33 (SD =1.58) years. Ethical approval was obtained from the School of Educational Sciences, Anshun University, China.

### Measures

### Negative life events

Developed by Liu et al. (1997), the adolescent life events scale consists of 27 items. The scale uses a 5-point Likert response scale. The scale comprises six subscales: interpersonal, academic stress, punishment, loss, health adaptation, and others. Sample items are as follows: “Admission pressure.” The scale has a good internal consistency with a Cronbach coefficient of 0.89.

### Adjustment

Developed by Fang et al. (2005), the Chinese college students’ adjustment scale comprises 60 items, including seven subscales: interpersonal adaptation, academic adaptation, campus life adaptation, career choice adaptation, emotional adaptation, self-adaptation, and satisfaction. The scale uses a Likert response ranging from 1 (“*disagree*”) to 5 (“*agree*”). Sample items are as follows: “I adapt very well to college life.” The scale has good reliability, with an internal consistency coefficient of 0.93.

### Self-esteem

The Rosenberg self-esteem inventory scale was developed by Rosenberg (1965) and revised by Yang and Wang (2007). The scale comprises 10 items on a 4-point scale. Sample items are as follows: “I’m optimistic about myself.” The higher the score, the higher the level of self-esteem. In the present study, the internal consistency coefficient of the scale was 0.88.

### Data collection and analysis

The investigators were postgraduate and undergraduate students. All the investigators were trained and qualified before conducting the survey. Before the survey, the participants read and signed the informed consent. It takes about 30 min to complete the survey for participants.

Statistical analyses were performed using SPSS 27.0. First, Harman’s single-factor test was conducted to assess the common method bias (Aguirre-Urreta and Hu, 2019). The result showed that there were 11 factors with characteristic roots greater than 1. The variation explained by the first factor was 27.35%, which was less than the critical value of 40%; therefore, there was no significant common method bias in this study. Second, Pearson correlation analyses were conducted to examine the relationships among all variables. Finally, we used PROCESS macro for SPSS to examine the moderated mediated analyses (Model 5) (Hayes, 2018). The independent variable was negative life events (X); the mediating variable was self-esteem (M); the dependent variable was adjustment (Y); the moderating variable was. The variable of grade were dummy coded (grade: freshman = 0, sophomore = 1, junior = 2, senior = 3). Bias-corrected bootstrap confidence intervals (CIs) were calculated with a yield of 95%. If the CI excluded zero, the moderated mediation model was significant.

## Results

### The descriptive statistics and correlation analysis

As Table 1 shows, negative life events were significantly negatively related to self-esteem (*r* = −0.24, *p* < 0.001) and college students’ adjustment (*r* = −0.37, *p* < 0.001) whereas self-esteem was significantly positively related to college students’ adjustment (*r* = 0.54, *p* < 0.001). The grade was significantly correlated with negative life events (*r* = − 0.06, *p* = 0.01), college students’ adjustment (*r* = 0.19, *p* < 0.001), and self-esteem (*r* = 0.06, *p* = 0.01).

**Table 1:** Descriptive statistics and Pearson correlations matrix (*n* = 1,717).

Variables	*M ± SD*	1	2	3	4	5	6
1.Age	21.33 ± 1.58	1.00					
2.Gender	*——*	0.13^**^	1.00				
3.Grade	*——*	0.70^**^	−0.04	1.00			
4.Negative life events	1.29 ± 0.57	0.02	0.05^*^	−0.06^*^	1.00		
5.College students’ adjustment	3.57 ± 0.73	0.17^**^	0.09^**^	0.19^**^	−0.37^***^	1.00	
6.Self-esteem	2.99 ± 0.43	0.01	0.11^**^	0.06^*^	−0.24^***^	0.54^***^	1.00

M, mean; SD, standard deviation. *p < 0.05, **p < 0.01, ***p < 0.001.

### The moderated mediation model

**Figure 1:**
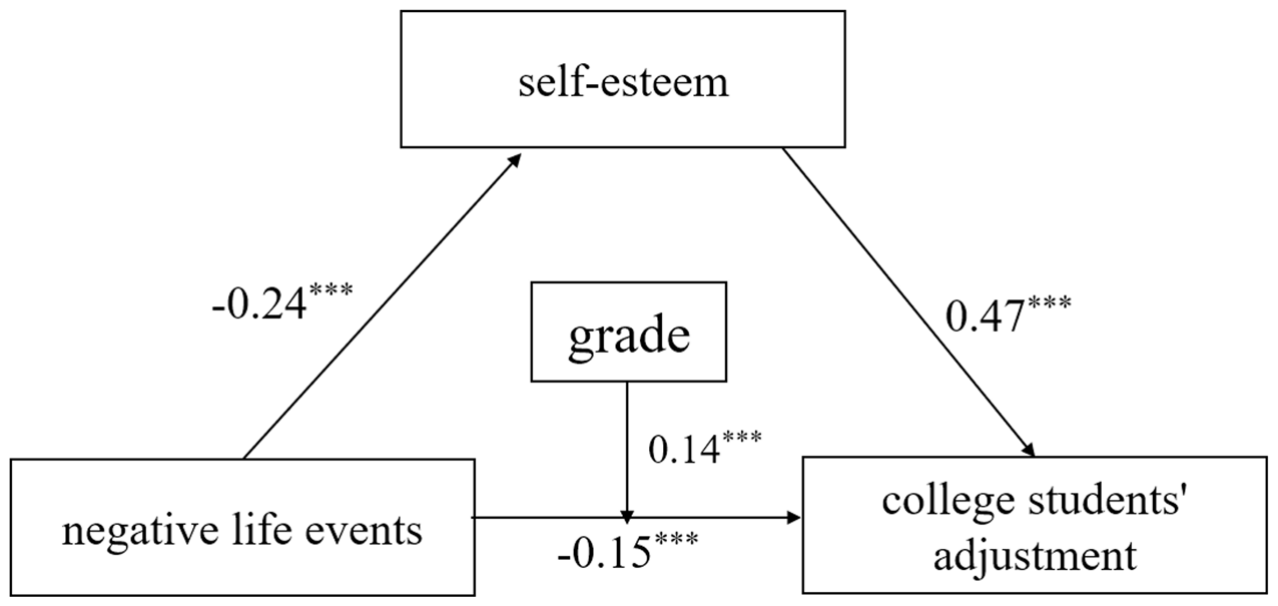
The moderated mediation model (*n* = 1,717). ***p < 0.001.

Two models were conducted to assess the moderated mediation of self-esteem and grade in the association between negative life events and college students’ adjustment (see Table 2 and Figure 1). The results found that the predictive effect of negative life events on self-esteem was significant in Model 1 (*β* = −0.24, *t* = −10.15, *p* < 0.001), indicating that the higher the effect of negative life events, the lower the level of self-esteem of college students. In Model 2, negative life events were a significant predictor of college students’ adjustment (*β* = −0.15, *t* = −7.22, *p* < 0.001) and self-esteem was a significant predictor of college students’ adjustment (*β* = 0.47, *t* = 24.11, *p* < 0.001), indicating that self-esteem mediates the relationship between negative life events and college students’ adjustment. The interaction term between negative life events and grade had a significant effect on college students’ adjustment (*β* = −0.04, *t* = −2.54, *p* < 0.01), indicating that grade plays a moderating role in the negative life events on college students’ adjustment.

**Table 2:** The moderated mediation models of self-esteem and grade in the relationship between negative life events and college students’ adjustment (*n* = 1,717).

Variables	Model 1 (outcome variable:self-esteem)			Model 2 (outcome variable:college students’ adjustment)		
	*β*	*SE*	*t*	*β*	*SE*	*t*
Negative life events	−0.24	0.024	−10.15^***^	−0.15	0.05	−3.18^***^
Self-esteem				0.47	0.02	24.11^***^
Grade				0.14	0.02	7.89^***^
Negative life events × Grade				−0.04	0.02	−2.54^*^
*R*^2^	0.06			0.38		
*F*	103.06^***^			264.99^***^		

*p < 0.05, **p < 0.01, ***p < 0.001.

The analysis of the mediated effect of negative life events on college students’ adjustment showed that the direct effect of negative life events on college students’ adjustment was 0.47 with a 95% confidence interval of [0.43, 0.51]. The effect values and confidence intervals for the effect of negative life events on college students’ adjustment at different grades are shown in Table 3.

**Table 3:** Moderating effect of grade on the relationship between negative life events and college students’ adjustment (*n* = 1,717).

Grade	Effect	Boot SE	BootLLCI	BootULCI
Freshman	−0.19	0.03	−0.25	−0.13
Sophomore	−0.23	0.02	−0.27	−0.19
Junior	−0.27	0.02	−0.32	−0.23
Senior	−0.32	0.03	−0.38	−0.25

**Figure 2:**
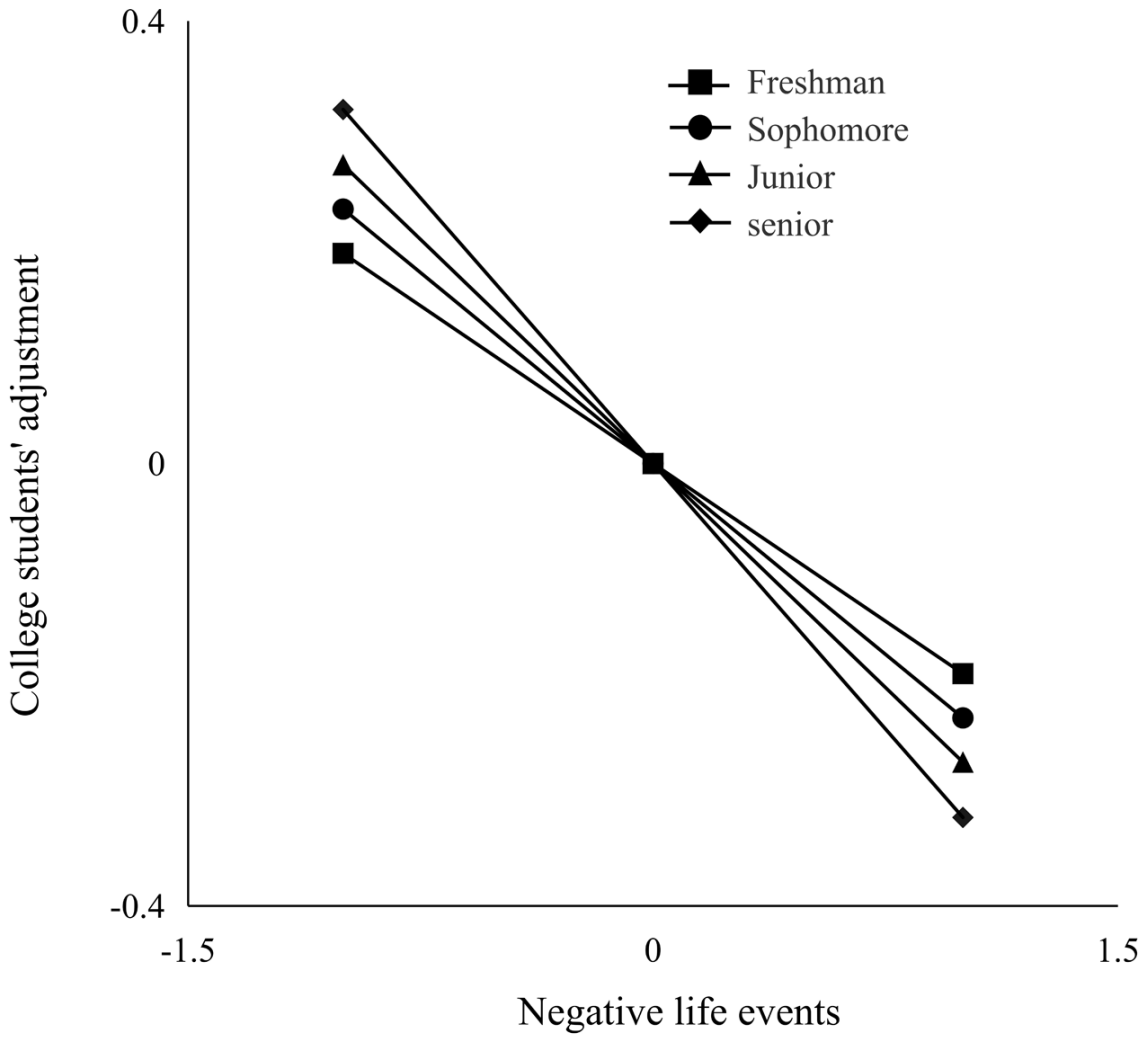
The moderated effect of grade on the relationship between negative life events and college students’ adjustment.

To further explore the moderating effect of grades in the relationship between negative life events and college students’ adjustment, we conducted a simple slope analysis and analyzed the effects of negative life events on college students’ adjustment under different grades (see Figure 2). The result found that the negative predictive effect of negative life events on college students’ adjustment was more significant among seniors compared to freshmen, sophomores, and juniors.

## Discussion

The results showed that there were significant correlations between negative life events, self-esteem, and college students’ adjustment, which are generally consistent with the findings of previous studies (Hou et al., 2017; Ma et al., 2020). There is a significant negative correlation between negative life events and self-esteem, indicating that many negative life events affect students’ self-esteem to some extent (Wan et al., 2019; Lee, 2020). It may be because negative life events reduce individuals’ evaluation of their own value and ability, thus forming low self-esteem. Individuals with low self-esteem lack a sense of control over themselves, thus resulting in low self-worth (Gao et al., 2020). Negative life events, as a source of social stress, are closely related to college students’ adjustment. Negative life events are negatively correlated with college students’ adaptation, which is consistent with previous research results (Lee, 2020; Sârbu et al., 2022). According to the life history theory, individuals who experience many negative life events are more likely to exhibit aggressive, antisocial, externalizing problem behaviors and poor academic performance, etc., and thus find it difficult to adapt to school life (Guo et al., 2005; Chang and Lu, 2018; Chang et al., 2019) Additionally, self-esteem is significantly and positively correlated with college students’ adjustment, indicating that higher levels of self-esteem in college students lead to better adaptation to college life. This can be attributed to the fact that students with high self-esteem can make positive, objective and correct evaluations and have a good understanding of themselves. They tend to be confident, optimistic, and able to appreciate the support, care, and encouragement from teachers and classmates, and love life and study more (Song et al., 2020). Consequently, they acquire recognition and trust, which enables them to adapt quickly to the college environment. In contrast, college students with low self-esteem tend to underestimate their abilities, lack confidence in accomplishing tasks, focus excessively on external perception and approval, experience negative emotions such as low self-esteem and depression due to frustrations, and struggle to communicate effectively and harmoniously with teachers and classmates. Consequently, they face difficulties in adapting well to college life.

The study findings reveal that self-esteem plays a mediating role in the relationship between negative life events and college students’ adjustment. This is consistent with previous studies (An et al., 2022). It is evident that negative life events directly impact college students’ adjustment and indirectly affect it through self-esteem. Individuals with high levels of self-esteem have the ability to buffer stressors from the outside world and can better adapt to college life (Kinnunen et al., 2008; Cui et al., 2021; Wang, 2021). Individuals with low self-esteem are more sensitive to external stimuli and pressure, and are prone to frustration. When facing negative life events, they tend to deal with problems in a way of fantasy or avoidance, constantly delay their actions, and appear academic procrastination behaviors, which lead to anxiety and depression, and ultimately lead to school maladjustment (Atteraya, 2021; Zhao, 2021). This study provides certain empirical and enlightenment for promoting college students’ adaptation. Although negative life events will have adverse effects on college students’ adaptation to a certain extent, such adverse effects will be gradually weakened through the mediation of self-esteem and positive regulatory effects, thus ensuring college students’ good adaptation (Han et al., 2018). Therefore, in the process of school education, improving the self-esteem level of college students has a certain role in promoting their good adaptation.

The study concluded that grade plays a moderating role in the effect of negative life events on college students’ adjustment. There are grade differences in the relationship between negative life events and college students’ adjustment. Specifically, with the increase of negative life events, the effects of negative life events on the adjustment is the greatest in freshman students, while the effects in senior students is relatively small. In other words, if the intensity of negative life events is reduced at the same time, the adjustment level of senior students is higher than that of freshman students, and negative life events have a greater effect on the adjustment of freshman students. The possible reasons are that for freshmen who have just entered the university, the difficulties were the transition from the original family environment dependent on parents to a relatively independent collective life, and the great change in living environment and lifestyle. Moreover, there are differences between university learning and middle school learning in learning purpose, learning content, learning style and learning requirements, and university emphasizes autonomous learning mode. These will make the new students think that they have encountered very difficult or feel uncomfortable (Zhang, 2020). Compared with freshmen, sophomores have become accustomed to college life and their interpersonal relationships are relatively stable. As for juniors, after 2 years of college life, they can control their emotions well, and they also know what they want and how they should work hard. For senior students, after the first 3 years of systematic knowledge learning and ability training, they have their own ways and methods in learning, familiar with the campus environment, stable interpersonal relationships, and the adjustment of college students has reached the best stage, which is in line with the law of personal growth and development (Feng, 2020). In sum, negative life events have a effect on college students’ adjustment, and students have a regulating effect on adjustment through continuous growth and development.

Finally, there are certain limitations in the present study that should be taken into consideration when interpreting the findings. Firstly, the study employed a cross-sectional design, which hinders the establishment of causality and makes it unclear how the relationship between negative life events and college students’ adjustment is influenced by self-esteem as a mediating factor and grade as a moderating factor. Secondly, while this study demonstrates the moderating effect of grades on the relationship between negative life events and college students’ adjustment, it is of utmost importance to investigate how these relationships evolve and which factors may exert influence. Therefore, further research is warranted.

## Conclusion

The study results provided support for the relationship between negative life events and college students’ adjustment. In the university, we should pay attention to the adverse effects of negative life events on students, and should minimize or avoid the occurrence and accumulation of negative life events. The school should focus on those students who encounter various changes in the short term, such as the death of relatives, parents divorce. The teachers should pay close attention to students’ adaptation, and provide psychological assistance in a timely manner. Moreover, it was found that self-esteem acted as a mediator in this relationship, while grades played a moderating role. These findings have significant implications for improving adjustment in Chinese college students. Considering the mediating effect of self-esteem, it is recommended to develop programs aimed at enhancing self-esteem in Chinese college students who experience high levels of negative life events, The teachers should give more affirmation and support to students with low self-esteem, help them improve their self-esteem level, and enhance their own psychological protection ability. Additionally, interventions targeting college students’ adjustment should consider the role of grades. For instance, schools should carry out entrance education for freshmen, explain the school history and school situation, let freshmen understand the development history and school philosophy of the school, so that freshmen have a preliminary understanding of the school at the beginning.

## Data availability statement

The raw data supporting the conclusions of this article will be made available by the authors, without undue reservation.

## Ethics statement

The studies involving humans were approved by the School of Educational Sciences, Anshun University, China (ID: ASU-JYXY-202208). The studies were conducted in accordance with the local legislation and institutional requirements. The participants provided their written informed consent to participate in this study.

## Author contributions

XQ: Writing – original draft, Conceptualization, Data curation, Formal analysis, Investigation, Methodology, Visualization. JS: Writing – review & editing, Conceptualization, Funding acquisition, Resources, Supervision.
